# Molecular Dynamics Approach to the Physical Mixture of In_2_O_3_ and ZrO_2_: Defect Formation and Ionic Diffusion

**DOI:** 10.3390/ijms24032426

**Published:** 2023-01-26

**Authors:** Lorenzo E. Fornasari, Bruna J. da S. Bronsato, Lucia G. Appel, Roberto R. de Avillez

**Affiliations:** 1 Departamento de Engenharia Química e de Materiais, Pontifícia Universidade Católica do Rio de Janeiro, PUC-Rio, Rua Marquês de São Vicente, 225, Gávea, Rio de Janeiro 22451-900, Brazil; 2 Divisão de Catálise e Processos Químicos, Instituto Nacional de Tecnologia, INT, Av. Venezuela 82/518, Rio de Janeiro 21081-312, Brazil

**Keywords:** In_2_O_3_, ZrO_2_, molecular dynamics, oxygen diffusion, epitaxy

## Abstract

Recent research on the use of physical mixtures In_2_O_3_-ZrO_2_ has raised interesting questions as to how their combination enhances catalytic activity and selectivity. Specifically, the relationship between oxygen diffusion and defect formation and the epitaxial tension in the mixture should be further investigated. In this study, we aim to clarify some of these relationships through a molecular dynamics approach. Various potentials for the two oxides are compared and selected to describe the physical mixture of In_2_O_3_ and ZrO_2_. Different configurations of each single crystal and their physical mixture are simulated, and oxygen defect formation and diffusion are measured and compared. Significant oxygen defect formation is found in both crystals. In_2_O_3_ seems to be stabilized by the mixture, while ZrO_2_ is destabilized. Similar results were found for the ZrO_2_ doping with In and ln_2_O_3_ doping with Zr. The results explain the high activity and selectivity catalyst activity of the mixture for the production of isobutylene from ethanol.

## 1. Introduction

Indium oxide is a versatile material that arouses interest in several applications, such as optoelectronics [1] and catalysis [2,3]. Recent researches have shown that the interest in this material for catalysis is due partially to the strong capacity of the cubic phase of In_2_O_3_ to form oxygen vacancies, especially in the presence of monoclinic ZrO_2_ [4,5,6,7]. The causes of this phenomenon are still not well understood and have been the target of theoretical and experimental studies for its elucidation. These materials seem to form an epitaxial alignment that favor defects’ formation, such as. oxygen vacancies, providing an interesting catalytic behavior [6,7]. 

Further, the oxygen dynamics of ZrO_2_ and In_2_O_3_ favor some reactions of industrial interest, like the syntheses of isobutene and acetone from ethanol [5,6] and also methanol and methane from CO_2_ [8,9]. These reactions depend on the oxygen lattice mobility, according to the Mars Van Krevelen mechanism [10].

The optimization and development of new In_2_O_3_ catalysts depend on understanding the phenomenon of point defects formation and mobility. In this sense, computer simulations help predict the behavior and properties of these materials. [11] Some authors have used Density Functional Theory (DFT) to investigate the morphology and stability of different faces of cubic In_2_O_3_ [12,13]. Walsh et al. [14] found that the stability of In_2_O_3_ surfaces can be described in the following order: (111) > (110) > (100). In addition, Zhang et al. [13] verified that the face (111) of In_2_O_3_ presents greater stability under oxidizing conditions, while the face (100) is more stable under oxygen-lean conditions. Through theoretical and experimental study, Ziemba et al. [15] used DFT calculations to simulate experimental Raman results and investigate the oxygen dynamics for cubic In_2_O_3_ under oxidizing and reducing conditions. From the calculations, they determined that the energy of formation of an oxygen vacancy in the bulk of an In_2_O_3_ unit cell was 3.40 eV, consistent with the values found in the literature [1,16]. In this study, the authors found that, under reducing conditions at temperatures above 120 °C, oxygen migrates from the bulk to the surface until it reaches an equilibrium condition, which is more favorable than maintaining oxygen vacancies on the surface of the oxide [15]. Nørskov et al. [17] calculated the energy of formation of oxygen vacancies on the surface (111) of In_2_O_3_ considering a supported catalyst model represented by In_2_O_3_ (111) layers on ZrO_2_ (111). The thickness of the In_2_O_3_ layer was varied (from 5 to 2 layers) to simulate different conditions of dispersion of In_2_O_3_ on the support. They found that reducing the In_2_O_3_ layer thickness increased the formation energy for oxygen vacancies, suggesting that increasing this oxide dispersion makes defect formation more difficult. For the more dispersed model (2 layers), the vacancy formation energy was 0 eV, while for the 5-layer model, the calculated value was −0.1 eV. The small vacancy formation energies show a relative stabilization of the In_2_O_3_ lattice in the physical mixture, resulting in a minor defect formation and a higher oxygen diffusion activation energy.

Molecular dynamics also contributed to the understanding and better description of materials but used empirical interatomic potentials to describe the structure of pure cubic In_2_O_3_ [1] or doped with elements as Sn [18,19]. Such potentials have often been used in molecular dynamics simulations to investigate the formation of defects as vacancies, Frenkel and Schottky [16,19,20], and to assess the stability of different faces of cubic In_2_O_3_ [14]. Warschkow et al. [18] proposed a Buckingham potential for In_2_O_3_, based on studies by Bush et al. [21]. Although this potential could provide adequate values for the structural properties of cubic In_2_O_3_, it barely fitted the dielectric parameters. Walsh et al. [1] reported a more accurate potential that simulates both the structural and physical properties of the material. Furthermore, such potential proved adequate for studies of intrinsic defects such as vacancies, Schottky, and Frenkel in this oxide. 

Recently, Hou et al. [19] developed a set of paired interatomic potentials, combining Buckingham, Lennard-Jones, Polynomial, Polynomial Harmonic Potentials, and Shell models. This new potential proved accurate to simulate structural and physical properties and understand the properties resulting from the formation of defects in the oxide. 

For ZrO_2_, most reported interatomic potentials are more appropriate for cubic or tetragonal phases and may present some deviations when used for simulations involving the monoclinic phase. Schelling et al. [22] developed a Buckingham potential to investigate the phenomenon of transition from cubic and tetragonal phases to cubic yttria-stabilized zirconia (YSZ). Kilo et al. [23] investigated oxygen diffusion in the same material over a finite temperature range, modifying the literature’s potentials. Lau & Dunlap [24] used diluted concentrations of Y_2_O_3_ in ZrO_2_ and described a suitable potential for the temperature range of 300–1400 K, using the rigid ion model approximation. Through a different line of study, Duin et al. [25] developed a ReaxFF reactive force field to search into the intergranular diffusion of perovskites like Y-doped BaZrO_3_ (BYZ). 

Despite these studies, there is not enough literature describing the interaction of In_2_O_3_ with ZrO_2_ based on molecular dynamics. Therefore, some questions are raised: would it be possible to use these potentials to describe the behavior of In_2_O_3_ and ZrO_2_ catalysts? Furthermore, how the presence of ZrO_2_ could affect the stability and oxygen mobility of In_2_O_3_?

This work uses molecular dynamics and empirical potentials to evaluate the interaction between In_2_O_3_ and ZrO_2_ oxides in proximity, the defect formation in the In_2_O_3_ oxide, and the diffusion of ionic species in mixtures of these oxides. The present results are discussed considering the known catalytic properties of the In_2_O_3_/ZrO_2_ system and its effectiveness in catalyzing the isobutylene synthesis from ethanol.

## 2. Methodology

The used interatomic potentials are a sum of a Coulomb term describing electrostatic interactions; and a Buckingham potential, which empirically describes the quantum interactions of the Pauli repulsion and the van der Waals attraction [26].

(1) $$V\left(r\right)=Aexp\left(\frac{-r_{ij}}{\rho }\right)-\frac{C}{r_{ij}^{6}}+\left(\frac{q_{i}q_{j}}{r_{ij}}\right)$$

where *r_ij_* is the distance between atoms *i* and *j*, *q_i_* and *q_j_* are their charges. The parameters *A*, *ρ*, and *C* were empirically derived.

The simulations were performed using the LAMMPS software, version 3 march 2020 [27] with the metal style. The cutoff radius was 10 Å for the Buckingham and 25 Å for the Coulomb interactions [1]. The long-range interactions were calculated using Ewald summations and long-range corrections were not used. The potentials’ parameters are presented in Table 1 with their respective references. These potentials presented the best match for oxygen-oxygen interactions and thus were best suited for combination. Further analysis on potential selection is presented in other sections of this work.

### 2.1. In_2_O_3_ Model Verification and Selection

This oxide’s crystal structure is described by a unitary cell containing 80 atoms and pertains to the Ia-3 spatial group [1]. The input files with the atomic positions for the rigid ion framework were obtained through a Python code using the Atomic Simulation Environment (ASE) library [28]. A simple Python algorithm produced the input files with the atomic positions for the core-shell framework from the rigid ion files.

Preliminary experiments were conducted on two existing potentials to determine which better describes the crystal. Furthermore, for each potential, a rigid ion and a core-shell case were investigated to check for a significant variation.

These experiments consisted of building a lattice parameter versus energy graph for each potential choice using a 4 × 4 × 4 In_2_O_3_ supercell. Runs for different lattice parameters were performed in NVT ensembles at room temperature for 4 ps using 0.2 fs time steps. The resultant medium potential energy per atom was registered (Figure S1). After that, a third-degree polynomial equation was fitted to the simulated points to help identify the local minimum which corresponds to the equilibrium lattice parameter. The bulk modulus, *B*, was extracted from the fitted polynomial through the following equation [29,30]:

(2) $$B=-V\frac{\partial P}{\partial V_{T}}=\frac{N}{9a_{0}}\;\frac{\partial^{2}E}{\partial a^{2}}$$

*V* is the volume at constant temperature, *P* is the pressure, *N* is the number of atoms in the unit cell, *a*_0_ is the equilibrium lattice parameter, *a* is the lattice parameter, and *E* is the potential energy described by the fitted third-degree polynomial equation.

For the linear expansion coefficient calculation, an NVT run with 4 ps of equilibration plus 0.2 ps steps of measurement was performed for different temperatures using a time step of 0.2 fs. The lattice parameter for each temperature was obtained and used to build a graph of elongation versus the difference in temperature to determine the linear expansion coefficient (Figure S2). 

The results were compared with experimental data and the original calculated values by their authors [1,18]. The adequate potential and case—core-shell or rigid ion—were chosen.

### 2.2. Defect Energy

The energies of various defects on the In_2_O_3_ crystal and its lattice energy were calculated and compared with Walsh et al. [1]. The rigid ion and the core-shell models were used, measured, and compared. Two different supercell sizes were considered: 3 × 3 × 3 and 4 × 4 × 4.

For each model and each supercell size, input files were crafted depicting the following cases: crystal with no defects; 8b indium atom vacancy; 24d indium atom vacancy; 48e oxygen atom vacancy; 8a interstitial indium atom; 16c interstitial indium atom; 24d interstitial indium atom; 16c interstitial oxygen atom; 24d interstitial oxygen atom; and 8a interstitial oxygen atom. The letters correspond to the Wyckoff positions [31,32]. Ten different input files with randomly positioned defects were generated for each defect type and interatomic potential. A minimization run was executed for each file, and the total energy average was calculated for each defect type. These values were subtracted from the total energy of the defect-free lattice with the same ion model and supercell size. 

For the Frenkel and Schottky defect energies, a calculation using the lower energy vacancies and interstitial atoms energies [1] was performed:

(3) $$E_{Schottky}=\frac{1}{5}\;\left(2E\;\left[V_{In}^{{}^{\prime }{}^{\prime }{}^{\prime }}\right]+3E\left[V_{O}^{\prime \prime }\right]\right)+E\left[In_{2}O_{3}\right])$$

(4) $$E_{Frenkel}=\frac{1}{2}\;\left(E\;\left[V_{In}^{{}^{\prime }{}^{\prime }{}^{\prime }}\right]+E\left[In_{i}^{{}^{\prime }{}^{\prime }{}^{\prime }}\right]\right)$$

(5) $$E\;\left[In_{2}O_{3}\right]=\frac{total\;potential\;energy}{N}\times 5$$

For the lattice energies, the total potential energy of the In_2_O_3_ crystal without defects was divided by the number of atoms, then multiplied by 5, the number of atoms in the In_2_O_3_ formula.

### 2.3. ZrO_2_ Model Selection

Bandura et al. compared different interatomic potentials to describe zirconia cubic, monoclinic and tetragonal phases [33]. Three interatomic potentials using the Buckingham potential were chosen for further analysis to allow compatibility with Walsh’s potential for In_2_O_3_, especially regarding the O^−2^-O^−2^ interaction, since the crystals were expected to exchange O^−2^ ions.

These candidate potentials did not have the monoclinic phase as their most stable. Instead presented an artificial rutile-like structure as their most stable phase. Stability tests were performed for these 3 potentials using 4 × 4 × 4 ZrO_2_ supercells, and NPT runs for 80 ps with 1fs time steps for stabilization plus 60 ps of measurement. The radial distribution functions of their zirconium atoms present in the stabilized phase were calculated and compared to the RDFs of other ZrO_2_ phases: artificial rutile-like, monoclinic and tetragonal. The cubic structure was not considered for it was the least stable phase for all 3 candidate potentials.

To further investigate compatibility between ZrO_2_ and In_2_O_3_ potentials, a graph of potential energy versus distance between oxygen ions was determined and compared.

### 2.4. Defective In_2_O_3_ and ZrO_2_

For the defective In_2_O_3_ experiments, 4 types of defects were investigated: Schottky defects, oxygen Frenkel defects, oxygen vacancies, and substitutional defects with Zr atoms occupying In sites. For defective ZrO_2_ experiments, Schottky defects, Frenkel defects, oxygen vacancies, and substitutional defects with In atoms occupying Zr sites. For each defect, input files representing different initial conditions were prepared using the ASE library.

A 3 × 3 × 3 supercell was used to investigate defects in In_2_O_3_. 13 Schottky defects were created by adding 26 In vacancies and 39 O vacancies to the base supercell. For Frenkel defects, 65 oxygen Frenkel defects were added to the supercell. Sixty-five oxygen atoms were eliminated from the supercell for pure oxygen vacancy defects. So the total number of vacancies was always 65, which is 3% of the total number of atoms in the perfect crystal.

The effect of Zr doping in the In_2_O_3_ crystal was investigated with two different files containing Zr doped In_2_O_3_. One had 78 Zr atoms, and another had 195 Zr atoms, substituted at random 8a In positions, as this is the indium atom site with the lowest vacancy formation energy. Vacancies for indium atoms were generated to equilibrate the crystals’ charge. Thus, the files with 78 Zr atoms contained the same number of In vacancies of the Schottky defect file, 26; and the files with 195 Zr atoms contained the same number of total vacancies of the Schottky defect file 65.

Similar files were generated to investigate defects on ZrO_2_ using a 5 × 5 × 5 base supercell. So to create 15 Schottky defects, 15 Zr vacancies and 30 O vacancies were created in the base supercell. For Frenkel defects, 45 oxygen Frenkel defects were added to the supercell. For pure oxygen vacancies, 45 oxygen atoms were taken from the supercell. So for each defect type, the total number of vacancies was 45, that is, 3% of the total number of atoms in the perfect crystal.

The effect of In doping in the ZrO_2_ crystal was investigated with two different files containing In using the 5 × 5 × 5 base supercell. One had 60 In atoms, and another had 90 In atoms, substituted at random Zr positions, as this crystal only possesses one type of site for Zr atoms. Vacancies for oxygen atoms were generated to equilibrate the crystals’ charge. Thus, the files with 60 In atoms contained the same number of oxygen vacancies of the Schottky defect file, 30; and the files with 90 In atoms contained the same number of total vacancies of the Schottky defect file, 45.

The defective crystals were built using the rigid model allowing an easier integration between different potentials. They were subject to a cg style hydrostatic pressure minimization at zero pressure, followed by a 50 ps equilibration hydrostatic NPT run at 1.01325 bar and a 1000 ps hydrostatic pressure NPT measuring run at various temperatures and 1.01325 bar. The simulation time and the time step of 1 fs were chosen to be similar to values used to simulate oxygen diffusion in Kilo et al. [23]. The temperatures were 850, 900, 950, and 1000 °C. The O^−2^-O^−2^ potential used for the doping simulations was that of the host crystal instead of Walsh’s potential for all O^−2^-O^−2^ interactions employed in the description of the physical mixtures. The mean square displacement of each atom type was computed during these experiments.

### 2.5. In_2_O_3_-ZrO_2_ Physical Mixture

**Figure 1:**
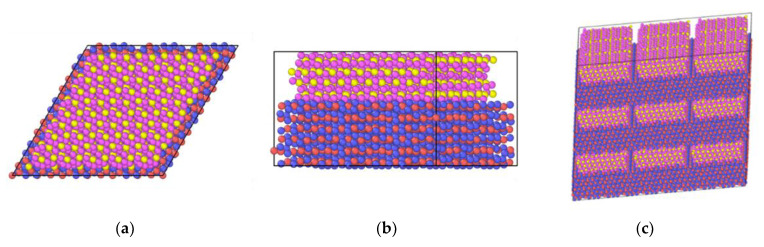
ZrO_2_(111) over In_2_O_3_(111): (**a**) top view, (**b**) front view, (**c**) ZrO_2_(111) over In_2_O_3_(111) with periodic structure replicated three times in each direction. Red is reserved for the In cations, with blue for oxygen in In_2_O_3_ structure; pink is oxygen, with yellow for Zr cation in ZrO_2_ slab.

Input files describing physical mixtures arrangements of layers of In_2_O_3_ and ZrO_2_ were crafted using the ASE library to generate slabs of each crystal in different planes and then processing these slabs with Python scripts to appropriately position the ZrO_2_ slabs on top of a larger In_2_O_3_ slab. As the ASE code outputted diamond-shaped slabs, this shape was conserved for the input data files. The used slabs were 3 × 3 × 3 In_2_O_3_(111), 5 × 5 × 4 ZrO_2_(111), 3 × 7 × 5 ZrO_2_(102), and 3 × 6 × 6 ZrO_2_(211). The 3 × 3 × 3 In_2_O_3_(111) slab dimensions were taken as a starting point to accommodate the ZrO_2_ chosen surface, and the ZrO_2_ slabs dimensions for the three different planes were chosen to cover most of the In_2_O_3_(111) slab surface; their length and width not being larger than the In_2_O_3_(111) slab surface; and the similarity in area and number of atoms between them. This last item is important because it allows a reasonable comparison between the different studied interfaces. The dimensions and number of atoms of the ZrO_2_ slabs are as follows: 5 × 5 × 4 ZrO_2_(111)—1,180,372 A^2^ and 1200 atoms; 3 × 7 × 5 ZrO_2_(102)—1266,318 A^2^ and 1260 atoms; 3 × 6 × 6 ZrO_2_(211)—1210,655 A and 1296 atoms. Figure 1a,b shows an example of the initial file used to simulate the physical mixture system with an In_2_O_3_ base. It is important to note that the generated input files have the In_2_O_3_ crystal touching the lateral edges of the periodic box, while this does not happen for the ZrO_2_ crystal present in these files. As this study uses periodic boundaries, which mirror the crystal structure in all directions, this leads to the formation of a continuous layer of the base crystal, leading to it experiencing a higher stabilization. Figure 1c shows the same system with the periodic structure replicated to show the formation of a continuous layer for the base oxide.

For each In_2_O_3_/ZrO_2_ combination, a twin file containing approximately 3% vacancies due to Schottky defects in each crystal was also crafted.

This file was manipulated into a rectangular box to fit LAMMPS limitations on simulation box angle and could not accept the box angle of the ZrO_2_ base used.

For comparison’s sake, a 6 × 6 × 5 ZrO_2_(111) slab was crafted as the base with its laterals touching the periodic boundary, and the 3 × 3 × 2 In_2_O_3_(111) slab placed on its top. If there are no significant differences between this slab’s arrangement and the previous, then the periodic boundary effect to stabilize the base crystal is insignificant. This setup file was manipulated into a rectangular box to fit LAMMPS limitations on simulation box angle and could not accept the box angle of the ZrO_2_ base used.

All files described were built using the rigid ion model. They were subject to a cg style triclinic minimization at 1.01325 bar, followed by a 50 ps equilibration triclinic NPT run at 1.01325 bar and a 1000 ps measuring triclinic NPT run at 850, 900, 950, 1000, and 1050 °C, and 1.01325 bar. The simulation time and the time step of 1 fs were chosen to be similar to values used to simulate oxygen diffusion in Kilo et al. [25]. During these experiments, the following information was acquired: the potential energy of each atom; the mean square displacement of each atom type, distinguishing the ZrO_2_ and In_2_O_3_ oxygen atoms; and the radial distribution function of each atom type.

### 2.6. In_2_O_3_-ZrO_2_ Interface 

Lewis [34] estimated the changes in cation coordination number on the repulsive term of the pair potential. They showed that the ionic radii for the tetrahedral site are about 0.94 the ionic radii for the octahedral site. This difference results in a reduction of 14% of the parameter A in Equation (1). The In_2_O_3_ and ZrO_2_ structures used in the present calculation have the same coordination number, so the same Zr^+4^-O^−2^ and In^+3^-O^−2^ pair potentials may be used inside the In_2_O_3_ and ZrO_2_ structures. Concerning the metallic ions at the interface region, one expects that the coordination number stays mostly the same since the oxygen ions must keep the electric neutrality of the crystal. Therefore, the same Zr^+4^ -O^−2^ and In^+3^-O^−2^ pair potentials were used for both crystals and the interface. Following Lewis [34], the O^−2^ -O^−2^ interaction is the same throughout the mixture, and the cation-cation interaction is purely Coulombic.

Before the mixture simulations, the chosen In_2_O_3_ and ZrO_2_ interfaces were placed at a short distance from each other, and the system was allowed to relax in all directions at 1.01325 bar (1 atm) and the desired temperature with a maximum volume change of 0.001 in any one direction at each interaction. After the equilibration, the timestep was reset, and an initial equilibration was run for 50,000 steps before the final simulation was run for 1,000,000 steps.

### 2.7. In_2_O_3_-ZrO_2_ Physical Mixture—ZrO_2_ Monolayer

ZrO_2_ was also simulated as a monolayer over the In_2_O_3_ crystal. Three different surface planes over an In_2_O_3_ crystal were crafted. The In_2_O_3_ dimensions were the same as before but the ZrO_2_ height corresponded to a unit cell perpendicular to the chosen plane. The number of atoms for each monolayer is ZrO_2_(111)—300 atoms; ZrO_2_(102)—252 atoms; and ZrO_2_(211)—324 atoms.

**Figure 2:**
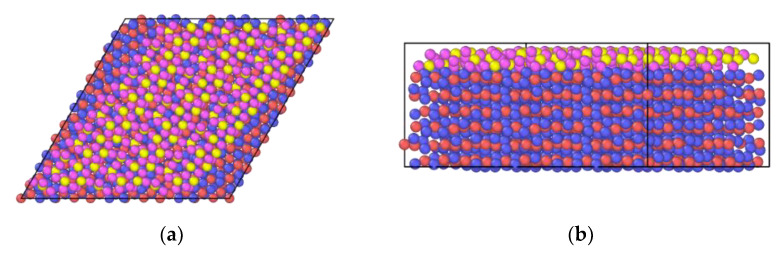
ZrO_2_(211) monolayer over In_2_O_3_(111): (**a**) top view, (**b**) front view.

For each In_2_O_3_/ZrO_2_(monolayer) combination, a twin file containing approximately 3% vacancies due to Schottky defects in each crystal was also crafted. Figure 2 shows an example of the In_2_O_3_/ZrO_2_(monolayer) combination.

All of the files were built using the rigid ion model. The files for the monolayers were subjected to the same simulation script used on the bulk physical mixtures.

### 2.8. MSD and Diffusion

The diffusion coefficient was computed from the atomic mean square displacement (MSD) determined every 100 timesteps for each group of atoms. The *MSD* shows a transient region that was not considered, and the stationary region was fitted with a linear function. The slope was used to calculate the diffusivity as a function of temperature, according to the following equations [35]:

(6) $$MSD=r_{i}^{2}\left(t\right)=\frac{1}{N}\;\overset{N}{\underset{i=1}{\sum }}{\left(r_{i}\left(t\right)-r_{i}\left(0\right)\right)}^{2}=6Dt\left(t\;\to \infty \right)$$

where *r_i_*(*t*) is the position of the atom *i* at the time *t*, *D* is the diffusion coefficient, *t* is the time.

The diffusivity, *D*, and the activation energy, *E_A_*, were obtained according to the following equations:

(7) $$D=D_{0}exp\left(\frac{-E_{A}}{RT}\right)$$

(8) $$ln\left(D\right)=ln(D_{0})-\frac{E_{A}}{RT}$$

Due to the relatively small equilibration run and low temperatures studied, many MSD curves only stabilized to a linear-like shape at some later point during the measurement run. Thus, the function for determining diffusivity was only fitted during this interval. For all other MSD curves that presented a linear shape from the beginning of the measurement run, the diffusivity function was fitted from steps 200 thousand to 1 million.

### 2.9. Defect Formation

The total number of interstitial oxygen atoms was also studied in the simulation runs of defective In_2_O_3_, defective ZrO_2_, and all physical mixtures at the lowest measured temperature, 850 °C. Because many studied cases already contained vacancies from the beginning of the simulation, only oxygen interstitials were measured to determine the oxygen vacancy formation through Frenkel defects in each system in a comparable manner. The time series and time averaging functions of the visualization software OVITO [36] were used to average the number of interstitial atoms over the total number of state ‘snapshots’ (LAMMPS dumps) taken after the stabilization of the system. The average interstitial count was divided by the total number of oxygen sites in the perfect crystal of the corresponding In_2_O_3_ or ZrO_2_ slab to normalize the different crystal sizes.

As with the MSD function fitting, the average of interstitial oxygen atoms was taken from step 200 thousand to one million as a rule of thumb. For systems in which MSD stabilized only later in the measurement run, this average was taken in the same interval used for the diffusivity function fitting.

### 2.10. Radial Distribution Function (RDF)

In some simulation runs where the coordination of atoms was measured, radial distribution function (RDF) measures were computed for that atom group through a LAMMPS compute every 200 timesteps. These measurements were then outputted every 20 thousand steps and analyzed through a Python script, producing graphs of RDF vs distance from the atom. The first undulation represents the first coordination shell of the atom, the first neighbors. The area under this first coordination shell is the atom’s coordination number. The coordination number of the first coordination shell was taken directly from the LAMMPS compute.

## 3. Results and Discussion

### 3.1. In_2_O_3_ Model Verification and Selection

The rigid ion model with Walsh’s potential provided the best description for the lattice parameter and the linear expansion coefficient experimental values, as evidenced in Table 2. So it was chosen for the present calculations. Lau & Dunlap [24] had already argued in their work that this approach is appropriate in MD simulations when no electric field is applied to the solid. The choice of the rigid ion model simplifies the integration between different Buckingham potentials. Table 3 shows all considered potential parameters for each crystal.

### 3.2. In_2_O_3_ Defect Energy

Table 4 shows that for both the 3 × 3 × 3 and the 4 × 4 × 4 supercell, the rigid ion model was overall more accurate when compared to the data presented by Walsh. The small differences between our simulations and Walsh’s results indicate that the supercell method used by us and the Mott-Littleton approach used by Walsh are comparable, which further validates the proposed rigid ion model.

### 3.3. ZrO_2_ Model Selection

The results presented in the literature for the various ZrO_2_ potentials [33] were organized to represent only those potentials capable of accurately modeling the ZrO_2_ crystal through the rigid-ion model. Table 5 compares candidate potentials for describing the ZrO_2_ crystal. The properties evaluated are the lattice parameters a, b, and c; the unit cell angle β; and the bulk modulus. This analysis concluded that the best three candidate potentials were Schelling’s, Lau’s, and Kilo’s.

**Figure 3:**
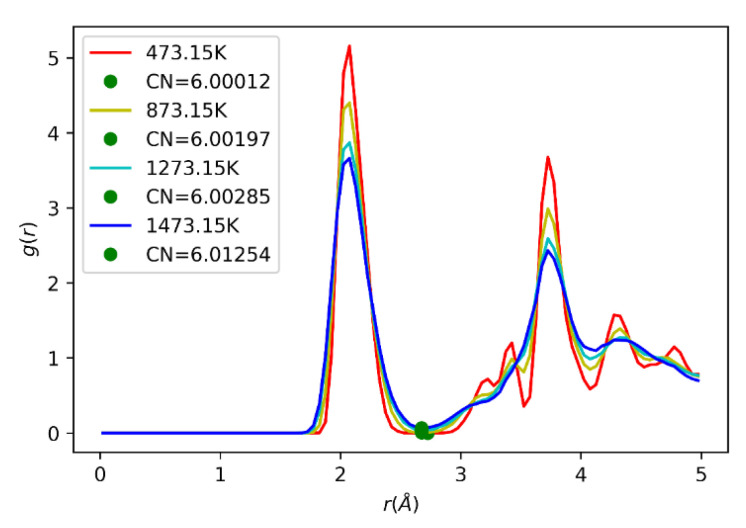
Zirconium radial distribution function as modeled by Kilo’s potential.

**Figure 4:**
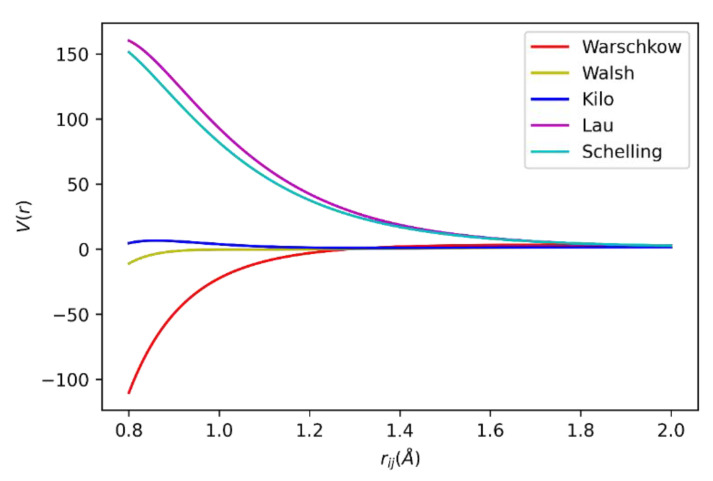
Oxygen-oxygen ion potential by analyzed models.

It was found that indeed none of them presents the monoclinic phase under the proposed simulated conditions. In the temperature range 473.15 K to 1273.15 K, both Lau’s and Kilo’s potential presented a zirconium coordination number of 6, as shown in Figure 3, while Schelling’s coordination number for zirconium was 8. Thus, it can be concluded that Lau’s and Kilo’s potentials developed an orthorhombic, rutile-like cell structure, while Schelling’s potential presented a tetragonal structure. Kilo’s potential was chosen to describe the ZrO_2_ compound. It results in an orthorhombic structure with a small deviation from the true monoclinic structure (less than 10 degrees in beta) and has an O^−2^–O^−2^ potential that resembles Walsh’s potential used to describe the In_2_O_3_ crystal, as shown in Figure 4. The similarity of the O^−2^–O^−2^ potentials suggests the use of only one potential description for both In_2_O_3_ and ZrO_2_ phases. When comparing the potential energy for oxygen ions interaction for the three best ZrO_2_ potentials and the two In_2_O_3_ potentials, it is clear that the potentials of Walsh and Kilo show great compatibility. So, Walsh’s potential was chosen to describe anionic interactions in the physical mixtures, as noted in Table 1.

### 3.4. Defective In_2_O_3_ and ZrO_2_

Table 6 shows the activation energy for oxygen diffusion considering different defects in both crystals. First, oxygen exhibits higher diffusion activation energy in ZrO_2_ for all intrinsic defects, probably due to the smaller interstitial size in this crystal. It is also observed that the diffusion activation energy increases following the same sequence of intrinsic defects for both the zirconia and indium oxide. Schottky has the lowest energy, followed by Frenkel and oxygen vacancies. To understand this phenomenon, OVITO was used. It was found that oxygen vacancies tended to distribute themselves symmetrically due to their effective repelling charges, which seem to stabilize the lattice and prevent diffusion to an extent. For Frenkel defects, it was observed that interstitial atoms tended to recombine with vacancies during the equilibration run. This way, by the time the measurement was done, there were significantly fewer Frenkel defects, and the crystal was almost perfect, thus making diffusion more difficult.

**Figure 5:**
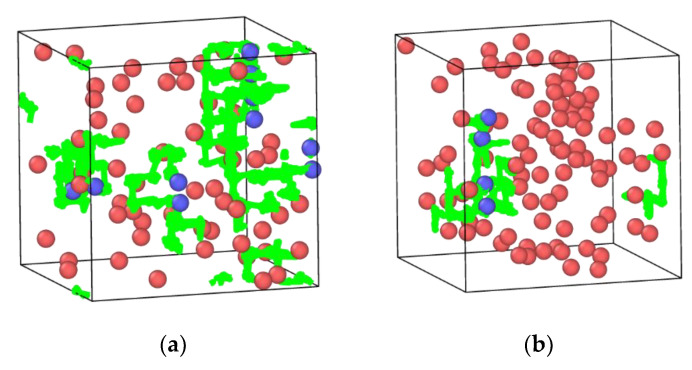
Substitutional indium atoms in red, interstitial oxygen atoms in the last data dump of the simulation in blue, and the diffusion paths of those oxygen atoms in green. (**a**) 60 substitutional In (**b**) 90 substitutional In.

**Figure 6:**
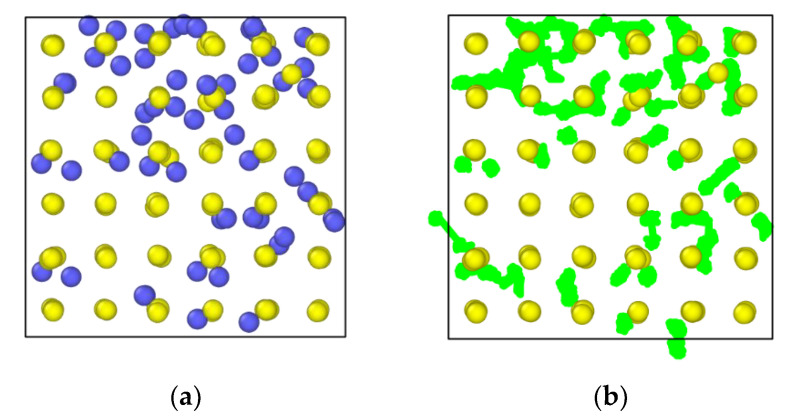
In_2_O_3_ doped with 22% percent zirconium. (**a**) Substitutional zirconium atoms in yellow, interstitial oxygen atoms in the last data dump of the simulation in blue, (**b**) the diffusion paths of those oxygen atoms in green.

Doped ZrO_2_ exhibits the lowest diffusion activation energy for the substitutional defects, while doped In_2_O_3_ oxygen exhibits the highest, which may be attributed to the type of defect sites generated by the doping and their effective charges. In doped ZrO_2_ generates oxygen vacancies and substitutional indium sites. Oxygen vacancies are more susceptible to aid in diffusion than cationic vacancies due to their effective positive charge and their matching size with the oxygen atom. Also, the effective negative charge of the substitutional indium atoms repels oxygen, avoiding trapping the anions in this way. Figure 5 shows the path of some interstitial oxygen atoms compared with the substitutional indium atoms position, showing that these cations tend to be avoided by diffusing anions. These phenomena help lower the diffusion activation energy for oxygen in zirconia. However, Zr doped In_2_O_3_ creates indium vacancies and substitutional zirconium sites, making oxygen diffusion more energetically costly. The created indium vacancies are larger than oxygen atoms and have an effective charge of −3, making them repel oxygen atoms, thus not favoring their diffusion. Further, substitutional zirconium atoms have an effective charge of +1, attracting and trapping oxygen atoms in their vicinity, and slowing their diffusion. Figure 6 shows the trapping effect of Zr substitutional atoms on oxygen diffusion.

(9) $$In_{2}O_{3}\;\to 2In_{Zr}^{\prime }+3O_{O}^{x}+V_{O}^{\prime \prime }$$

(10) $$3ZrO_{2}\;\to 3Zr_{In}^{\prime }+6O_{O}^{x}+V_{In}^{{}^{\prime }{}^{\prime }{}^{\prime }}$$

The data for oxygen defect formation in Table 7 does not show many interesting patterns. The ZrO_2_ crystal showed higher overall defect formation. Also, as mentioned before, the recombination of interstitials and vacancies during the equilibration step reduces the number of Frenkel defects by the time of the measurement run. No obvious correlation between diffusion activation energy and defect formation was identified.

### 3.5. In_2_O_3_-ZrO_2_ Physical Mixture

Table 8 shows the oxygen diffusion activation energy with positive values for all attempted combinations. The mean square displacement of cations was very low, and for this reason, their diffusion activation energies were not calculated (Figure S3). It should also be noted that some calculations have near-zero or even negative oxygen diffusion activation energies due to the very low diffusivity (Figures S4 and S5). It is believed that the computation time was too short or the temperature too low.

Table 8 presents interesting trends. In all physical mixtures, the activation energy for oxygen diffusion in no-defects indium oxide was greater than the value observed for the 3% Schottky non-mixture In_2_O_3_ crystal (Table 6). The physical mixture shows the same qualitative effect on oxygen diffusion activation energy as the doping of these oxides with one another—ZrO_2_ elevates oxygen activation energy in In_2_O_3_, and In_2_O_3_ lowers the oxygen activation energy in ZrO_2_. The activation energy for oxygen diffusion in no-defect zirconia in the physical mixture was lower than for all intrinsic defects in non-mixture ZrO_2_. However, the lowest energy for all ZrO_2_ oxygens was still found for the case of substitutional indium defects in a non-mixture ZrO_2_ crystal, which points to an important result.

**Figure 7:**
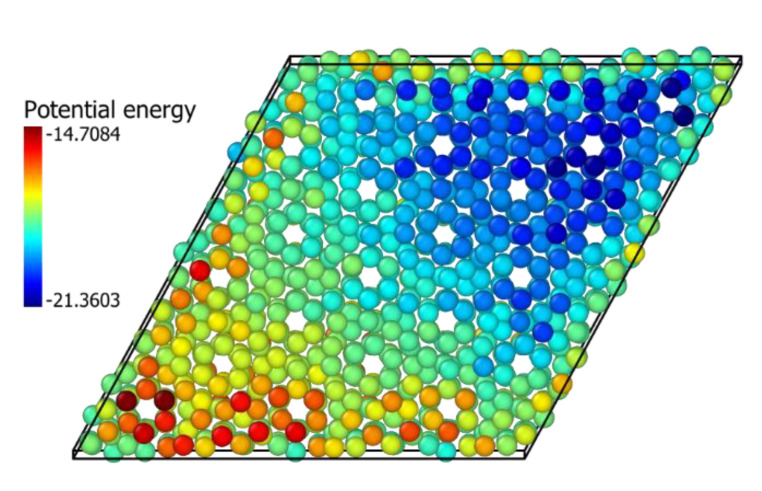
ZrO_2_(111) over In_2_O_3_(111) physical mixture. In_2_O_3_ oxygen atoms potential energy at the interface (eV).

**Figure 8:**
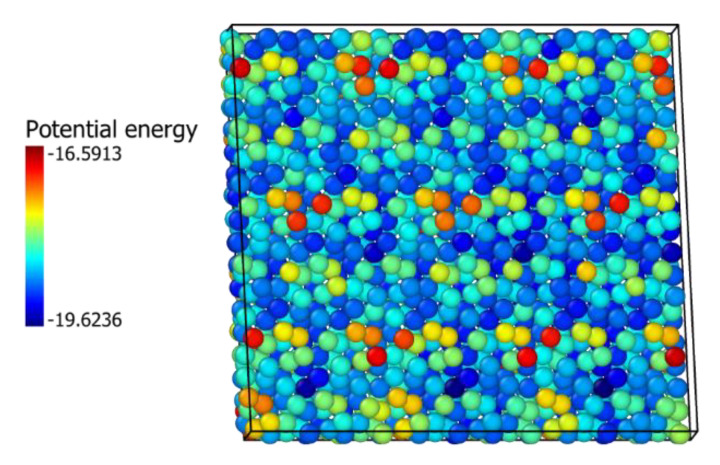
In_2_O_3_(111) over ZrO_2_(111) physical mixture. ZrO_2_ oxygen atoms potential energy at the interface (eV).

The mechanical, physical mixtures with the associate epitaxial piling of the ZrO_2_ and In_2_O_3_ nanocrystals affect oxygen diffusion equivalent to a doping process. It results from either small doping at the two materials interface or, most probably, the lattice tensions caused by the epitaxy between the two crystals. Yamamoto et al. [41] observed from DFT calculations that compressive stress caused a reduction in the oxygen diffusion coefficient in monoclinic and tetragonal ZrO2. These lattice tensions are illustrated in Figure 7 and Figure 8, which show how the interaction between crystals creates patterns of potential energies. For the In_2_O_3_ crystal, it seems these tensions stabilize oxygen atoms, inhibiting their diffusion even more than doping with zirconium, which agrees with the calculations from Nørskov et al. [17], that showed that oxygen vacancy formation energy is elevated in In_2_O_3_ by its contact with ZrO_2_. For ZrO_2_, the effect seems to be destabilization of the atom, although doping with indium still stimulates diffusion more significantly.

For the ZrO_2_ oxygens present in bulk ZrO_2_ over In_2_O_3_ and for In_2_O_3_ oxygens present in In_2_O_3_ over ZrO_2_, another interesting pattern is the lower diffusion activation energy of the setups without intrinsic defects in comparison to those with them. This behavior also points to the great influence of lattice tensions on oxygen diffusion in the studied physical mixtures. It is argued that for crystals with intrinsic defects, there is more room for lattice tensions to be dissipated through lattice distortion, while in perfect crystals there is not an easy outlet for this accumulated energy apart from atoms jumping lattice sites.

Lastly, activation energies for ZrO_2_ oxygens diffusion varied little between physical mixtures, presenting values between 0.51 and 0.88 eV, which is likely due to the stronger attraction oxygen atoms have towards zirconium atoms. 

The results for defect formation in physical mixtures in Table 9 do not show any obvious correlation between defect formation and diffusion activation energy. Nevertheless, some general statements can be made about them. For all cases, physical mixtures had higher defect formation than their respective crystals with Schottky defects. So the mixture promotes defect formation in both crystals. These results strengthen earlier rationalizations in catalysis, which linked the physical mixture’s catalytic activity to increased oxygen vacancies in the In_2_O_3_ lattice [5,6,7,9,17]. Beyond that, physical mixtures with Schottky defects exhibited higher Frenkel defect formation percentages when compared to their non-defective counterpart. For In_2_O_3_ as the substrate, a monolayer of ZrO_2_ increases defects formation in both crystals, although this effect is more pronounced in the crystal which forms the monolayer.

Moreover, the ZrO_2_ crystal tended to form more Frenkel defects, just as in the defective crystal cases, except when it was the base crystal with edges touching the periodic boundary. It suggests that whether or not a crystal’s edges touch the periodic boundary does have significant implications for the systems studied. It should also be noted that defect formation was disproportionately higher in ZrO_2_ in the cases where it was not the substrate crystal compared to In_2_O_3_ when it was not the substrate crystal. So, even if the stabilization effect of the periodic boundary is important, the interaction between ZrO_2_ and In_2_O_3_ plays a major role in promoting or limiting defect formation. More specifically, the interaction promotes more oxygen vacancies for the ZrO_2_ lattice more than for the In_2_O_3_. These results are in contrast to experimental evidence [5,7]. Nonetheless, the studies that yielded this experimental evidence used a much smaller weight ratio of indium to zirconium oxide when preparing their catalysts than the weight ratio of the simulated physical mixtures.

**Figure 9:**
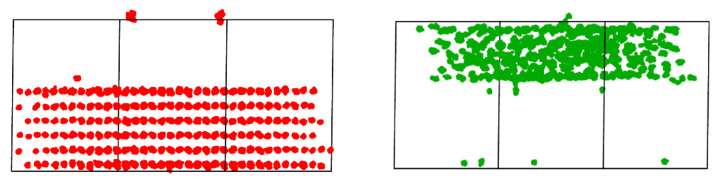
Indium (red) and zirconium (green) diffusion paths in defective ZrO_2_(102) over In_2_O_3_(111) physical mixture.

Although the diffusion of cations was not high enough to allow calculation of its activation energies, it was high enough to be detected by a simple visual inspection in OVITO, as shown in Figure 9. So Zr atoms in In_2_O_3_ have been found to stabilize it, thus limiting excessive vacancy formation on its surface [13]. Furthermore, the diffused cations would form a very diluted solution in the other crystal, with a concentration of approximately one in thousands in the receiving crystal, but this could have important implications for catalysis. Catalysts of ZrO_2_ doped by Zn in similar concentrations have already been found effective in catalyzing isobutylene synthesis from ethanol [42].

Though they do not constitute conclusive evidence, these observations about the zirconia crystal all point in the direction that it may have a more important role in the catalysis of ethanol to isobutylene than previously proposed when in a physical mixture with In_2_O_3_. Sun et al. [43] demonstrated in their theoretical DFT and experimental studies that the presence of ZrO_2_ played a key role in stabilizing the surface oxygen atoms of In_2_O_3_ in In_2_O_3_/ZrO_2_-based catalysts in the hydrogenation of CO_2_ to methanol. Our study provides complementary results, that ZrO_2_ may likewise play an important role in the reaction to obtain isobutene. Increased oxygen diffusivity and defect formation within the crystal are good indicators of its higher ability to contribute to the acetone generation via Mars Van Krevelen mechanism one important intermediate of the isobutene synthesis [10]. Besides that, the diffusion of indium atoms to the ZrO_2_ lattice is significant as the use of doped ZrO_2_ with another low valence dopant [42,44,45], Zn, has already been proved effective for the isobutylene synthesis from ethanol by acting on the rate-limiting step of the generation of acetone, intermediate of this olefin synthesis, from ethanol) [44,45]. Thus a complete understanding of this cascade reaction could involve both In_2_O_3_ and ZrO_2_ acting on different steps along its way. Nonetheless, the ZrO_2_-In_2_O_3_ system seems to follow the moderation principle suggested by McFarland [46] which states that the oxygen must not be easily removed from the catalyst surface since it would be difficult to have it back and close the Mars Van Krevelen mechanism.

## 4. Conclusions

The results regarding oxygen diffusion pointed out a distinct pattern whereby the physical mixture of one oxide on another had the same qualitative effect as their doping—the activation energy for oxygen diffusion was lowered in ZrO_2_ and became higher in In_2_O_3_. This behavior is most likely due to the lattice tensions generated by the epitaxy between the crystals.

Further, the modified physical mixture promotes oxygen Frenkel defect formation in both crystals at higher concentrations in the case of the ZrO_2_ crystal, which confirms the observations from past studies that oxygen vacancies formation is promoted in In_2_O_3_ by the presence of ZrO_2_.

The present calculations also indicate the presence of very small cation diffusion between both crystals in the physical mixture, which will change the defect concentration in the indium and zirconium oxides.

For the In_2_O_3_ crystal in the physical mixture, the present results matched well the vacancy formation, oxygen stabilization, and higher diffusion activation energy. In contrast, for the ZrO_2_ crystal in the modified physical mixture, higher overall oxygen vacancy formation, the lower activation energy for oxygen diffusion, and the diffusion of indium cations into its structure all pointed to a role not previously considered in the literature for this catalytic system. Furthermore, the changes observed for ZrO_2_ in our computational analyzes contrasted with some experimental data obtained previously. Indeed, published previous investigations show higher concentrations of ZrO_2_ than those of this work. Considering that the techniques usually employed in catalyst characterization do not show lateral resolution, it was not possible to observe these ZrO_2_ changes.

Thus, the physical mixture’s role as a catalyst is due to the role of two modified materials and the interactions resulting from the interdiffusion of In_2_O_3_ and ZrO_2_ crystals.

## Figures and Tables

**Table 1:** Buckingham interatomic pair potential parameters for the In_2_O_3_ and ZrO_2_ physical mixture.

	A (eV)	ρ (Å)	C (eV Å^6^)	Reference
In^+3^—O^−2^	1498.651	0.3405	33.2	[1]
Zr^+4^—O^−2^	1024.600	0.3760	0.0	[23]
O^−2^—O^−2^	22,764.000	0.1490	32.0	[1]

**Table 2:** Comparison of the different In_2_O_3_ interatomic potentials and experimental data.

		Walsh’s Potential			Warschkow’s Potential		
Properties	Experiment	Reference [1]	Core-Shell Model Results	Rigid Ion Model Results	Reference	Core-Shell Model Results	Rigid Ion Model Results
Lattice parameter (Å)	[37] 10.117	10.1210	10.0502	10.1188	10.1200	10.4186	10.1182
Bulk Modulus (GPa)	[38] 194.24	193.7700	204.9747	196.8721	222.7900	179.8271	226.5402
Linear expansion coefficient, α (°C^−1^)	[39] 6.15 × 10^−6^	NA	5.25 × 10^−6^	6.14 × 10^−6^	NA	1.25 × 10^−5^	7.22 × 10^−6^
	[40] 7.5 × 10^−6^						

**Table 3:** Interatomic potentials used for the present calculations.

	A (eV)	ρ (Å)	C (eV Å^6^)	Reference
In_2_O_3_ crystal				
In^+3^—O^−2^	1498.651	0.340500	33.2000	[1]
O^−2^—O^−2^	22,764.000	0.149000	32.0000	[1]
In^+3^—O^−2^	2719.770	0.291700	0.0000	[18]
O^−2^—O^−2^	25.410	0.693700	32.3200	[18]
ZrO_2_ crystal				
Zr^+4^—O^−2^	1024.600	0.376000	0.0000	[23]
O^−2^—O^−2^	22,764.000	0.149000	27.8900	[23]
Zr^+4^—O^−2^	1292.850	0.358388	19.3646	[24]
O^−2^—O^−2^	13,098.900	0.219670	49.2998	[24]
Zr^+4^—O^−2^	1502.110	0.3450	5.1000	[22]
O^−2^—O^−2^	9547.960	0.2240	32.0000	[22]

**Table 4:** In_2_O_3_ defect energies in eV.

		3 × 3 × 3 Supercell				4 × 4 × 4 Supercell			
	Reference	Core-Shell Model		Rigid Ion Model		Core-Shell Model		Rigid Ion Model	
Defect Type	Walsh et al., 2009 [1]	Simulated Values	Difference from Reference	Simulated Values	Difference from Reference	Simulated Values	Difference from Reference	Simulated Values	Difference from Reference
Lattice energy	−140.60	−141.40	0.6%	−140.57	0.0%	−141.40	0.6%	−140.57	0.0%
In vacancy (8b)	49.92	49.82	0.2%	50.08	0.3%	50.21	0.6%	50.31	0.8%
In vacancy (24d)	50.05	50.42	0.7%	50.99	1.9%	50.72	1.3%	51.22	2.3%
O vacancy	20.99	21.71	3.4%	21.13	0.7%	21.89	4.3%	21.23	1.1%
In intersticial (8a)	−33.57	−34.71	3.4%	−33.99	1.3%	−34.38	2.4%	−33.76	0.6%
In intersticial (16c)	−36.21	−35.75	1.3%	−35.48	2.0%	−35.41	2.2%	−35.27	2.6%
In intersticial (24d)	−34.89	−34.09	2.3%	−33.41	4.2%	−33.74	3.3%	−33.08	5.2%
O intersticial (8a)	−13.29	−14.20	6.8%	−13.13	1.2%	−14.03	5.6%	−13.03	2.0%
O intersticial (16c)	−14.61	−15.43	5.6%	−14.54	0.5%	−15.25	4.4%	−14.44	1.2%
O intersticial (24d)	−12.08	−13.03	7.9%	−11.70	3.1%	−12.84	6.3%	−11.60	4.0%
cation Frenkel pair	6.85	7.04	2.8%	7.40	8.0%	7.40	8.0%	6.48	5.4%
anion Frenkel pair	3.19	3.14	1.6%	3.30	3.4%	3.32	4.1%	3.43	7.5%
Schottky defect.	4.44	4.67	5.2%	4.58	3.2%	4.94	11.3%	4.71	6.1%
Differences mean			3.2%		2.3%		4.2%		3.0%

**Table 5:** Comparison of the different ZrO_2_ interatomic potentials and experimental data.

		Schelling		Kilo		Lewis-Catlow		Lau-Dunlap	
Properties	Experimental Values for monoclinic ZrO_2_	Simulated Values	Difference from Experiment	Simulated Values	Difference from Experiment	Simulated Values	Difference from Experiment	Simulated Values	Difference from Experiment
a (Å)	5.149	5.234	1.7%	5.291	2.8%	4.954	3.8%	5.201	1.0%
b (Å)	5.208	4.904	5.8%	4.973	4.5%	4.954	4.9%	4.871	6.5%
c (Å)	5.316	5.574	4.9%	5.624	5.8%	4.954	6.8%	5.564	4.7%
β (°)	99.23	90.00	9.3%	89.99	9.3%	90.00	9.3%	90.00	9.3%
Bulk modulus (Gpa)	201	235	16.9%	196	2.5%	327	62.7%	236	17.4%
Mean difference			7.7%		5.0%		17.5%		7.8%

**Table 6:** Oxygen diffusion activation energy (eV) in defective crystals. * Indicates that not all temperatures were used due to negative diffusivity being calculated in one of them.

Defects	Pseudo-Monoclinic ZrO_2_	Cubic In_2_O_3_
3% Schottky	1.0466	0.3626
Frenkel	1.5041 *	0.4065
Oxygen vacancies	2.4801	0.5114
12% substitutional In	0.3093	
18% substitutional In	0.4974	
9% substitutional Zr		1.3753
22% substitutional Zr		1.2548

**Table 7:** Anion Frenkel defects formation percentage at 850 °C in defective crystals.

Defects	Pseudo-Monoclinic ZrO_2_	Cubic In_2_O_3_
3% Schottky	0.87%	0.00%
Frenkel	0.42%	0.51%
Oxygen vacancies	1.19%	0.00%
12% substitutional In	0.54%	
18% substitutional In	0.45%	
9% substitutional Zr		0.67%
22% substitutional Zr		1.90%

**Table 8:** Oxygen diffusion activation energy (eV) in physical mixtures.

	In_2_O_3_ Oxygen		ZrO_2_ Oxygen	
Physical Mixture	No Defects	3% Schottky	No Defects	3% Schottky
Pseudo-monoclinic ZrO_2_(111) over cubic In_2_O_3_(111)	2.0224	0.4331	0.5101	0.8796
Pseudo-monoclinic ZrO_2_(102) over cubic In_2_O_3_(111)	0.9851	0.4600	0.6161	0.7661
ZrO_2_(211) monolayer over cubic In_2_O_3_(111)	1.0464	0.9555	0.7971	0.6970
Cubic In_2_O_3_(111) over pseudo-monoclinic ZrO_2_(111)	0.4262	0.8195	0.8433	0.8307

**Table 9:** Anion Frenkel defects formation percentage at 850 °C in physical mixtures.

	In_2_O_3_ Oxygen		ZrO_2_ Oxygen	
Physical Mixture	No Defects	3% Schottky	No Defects	3% Schottky
Pseudo-monoclinic ZrO_2_(111) over cubic In_2_O_3_(111)	1.02%	1.61%	11.55%	13.17%
Pseudo-monoclinic ZrO_2_(102) over cubic In_2_O_3_(111)	1.49%	2.08%	12.27%	14.87%
ZrO_2_(211) monolayer over cubic In_2_O_3_(111)	1.86%	2.46%	20.72%	22.73%
Cubic In_2_O_3_(111) over pseudo-monoclinic ZrO_2_(111)	5.89%	7.52%	3.19%	4.12%

## Data Availability

The LAMMPS and PYTHON codes are available upon request.

## Supplementary Materials

The following supporting information can be downloaded at: https://www.mdpi.com/article/10.3390/ijms24032426/s1, Figure S1: – average potential energy per atom vs lattice parameter graph for In_2_O_3_ described by Walsh’s rigid ion model; Figure S2: elongation vs difference in temperature for In_2_O_3_ described by Walsh’s rigid ion model; Figure S3: MSD over time for indium atoms in the ZrO_2_(111) over In_2_O_3_(111) physical mixture with no initial defects; Figure S4: MSD over time for In_2_O_3_ oxygen atoms in the ZrO_2_(211) over In_2_O_3_(111) physical mixture with no initial defects; Figure S5: ln(D) over T^-1^ for In_2_O_3_ oxygen atoms in the ZrO_2_(211) over In_2_O_3_(111) physical mixture with no initial defects.
