# Supplementary material for: Molecular Dynamics Approach to the Physical Mixture of In2O3 and ZrO2: Defect Formation and Ionic Diffusion
Source: Int J Mol Sci. 2023 Jan 26;24(3):2426. doi: 10.3390/ijms24032426 (PMC9917225; doi:10.3390/ijms24032426)
Supplement: Supplementary file 1 [file ijms-24-02426-s001.zip › ijms-2161744-supplementary.pdf]

Supplementary Material: Molecular Dynamics Approach to the Physical Mixture of In_2O_3 and ZrO_2: defect formation and ionic diffusion

Lorenzo E. Fornasari, Bruna J. da S. Bronsato, Lucia G. Appel, Roberto R. de Avillez

Introduction

Some of the results from the potential testing and physical mixtures simulations were omitted from the main paper for the sake of simplicity. Here we detail these results more deeply

In_2O_3 model verification and selection

Figure S1 shows the third-degree polynomial equation fitted to the values of lattice parameters and medium potential energy per atom simulated by Walsh's rigid ion model. The local minimum is identified by a green dot and represents the equilibrium lattice parameter. The polynomial represented by the blue line describes the value of *E* in equation 2 of the main paper.

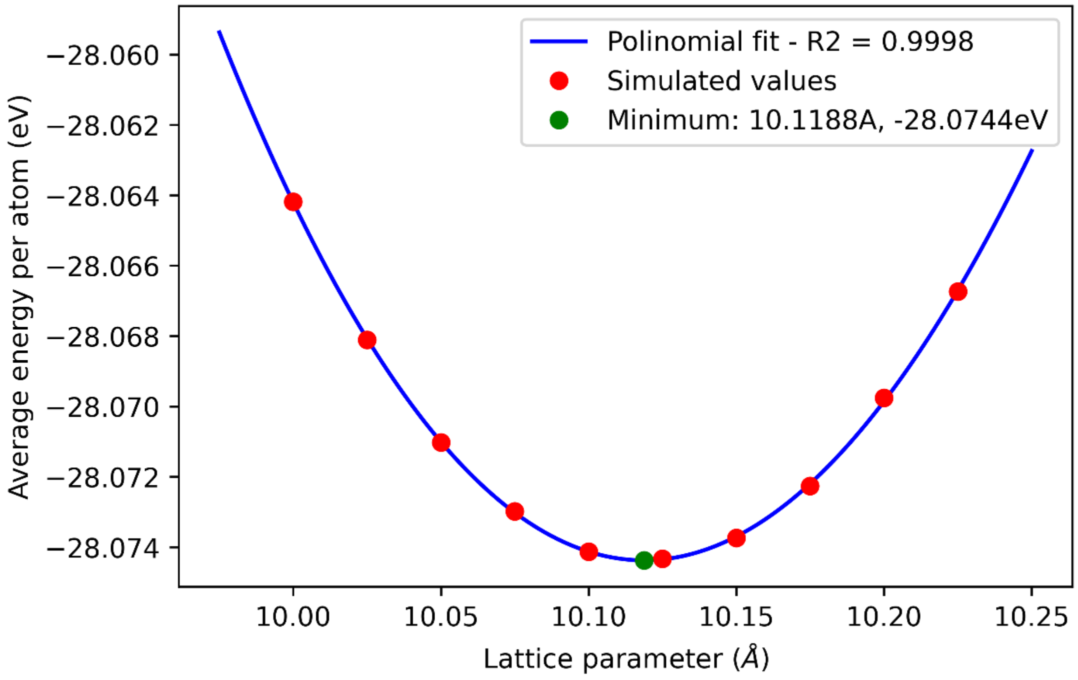

Figure S1 – average potential energy per atom vs lattice parameter graph for In_2O_3 described by Walsh's rigid ion model

Figure S2 shows the graph built to determine the linear expansion coefficient for Walsh's rigid ion model. A linear function is fitted to the values of elongation and difference in temperature from a reference case, then the slope is taken as the linear expansion coefficient.

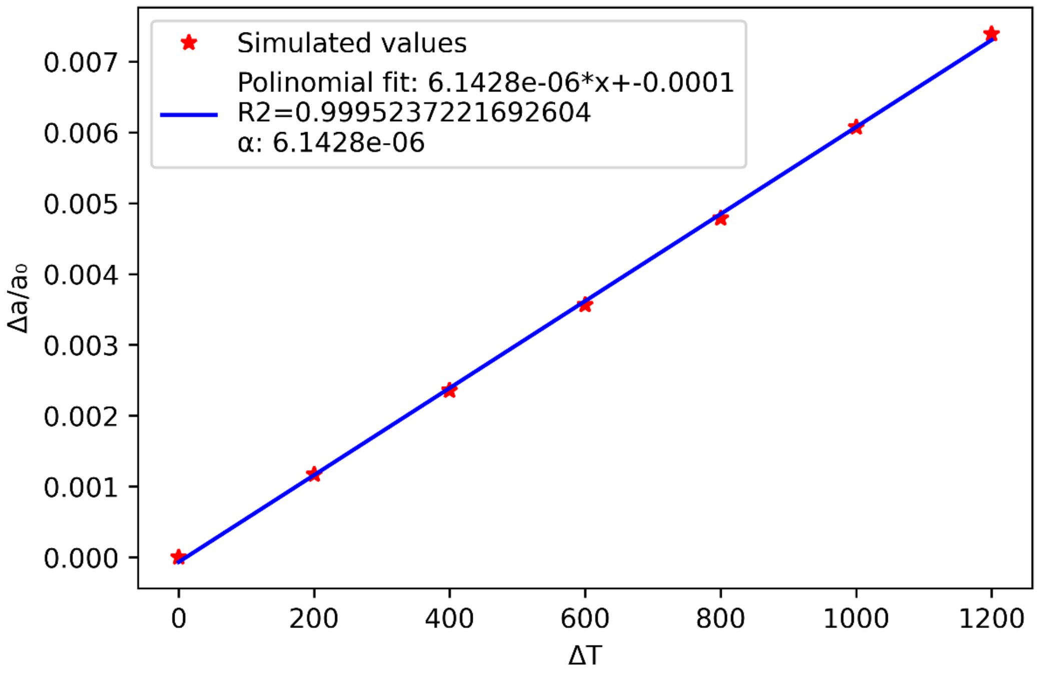

Figure S2 – elongation vs difference in temperature for In_2O_3 described by Walsh's rigid ion model

In_2O_3-ZrO_2 physical mixture

Figure S3 shows the fitting of a linear function to the values of MSD of indium atoms over time simulated for the ZrO_2(111) over In_2O_3(111) physical mixture with no initial defects. The fitted function is represented as the black line and the simulated values change in color for each simulated temperature. It can be seen that all lines show a near-constant value through time and that the measured diffusivity is very low. This was the general pattern for the simulated cations in all simulation runs.

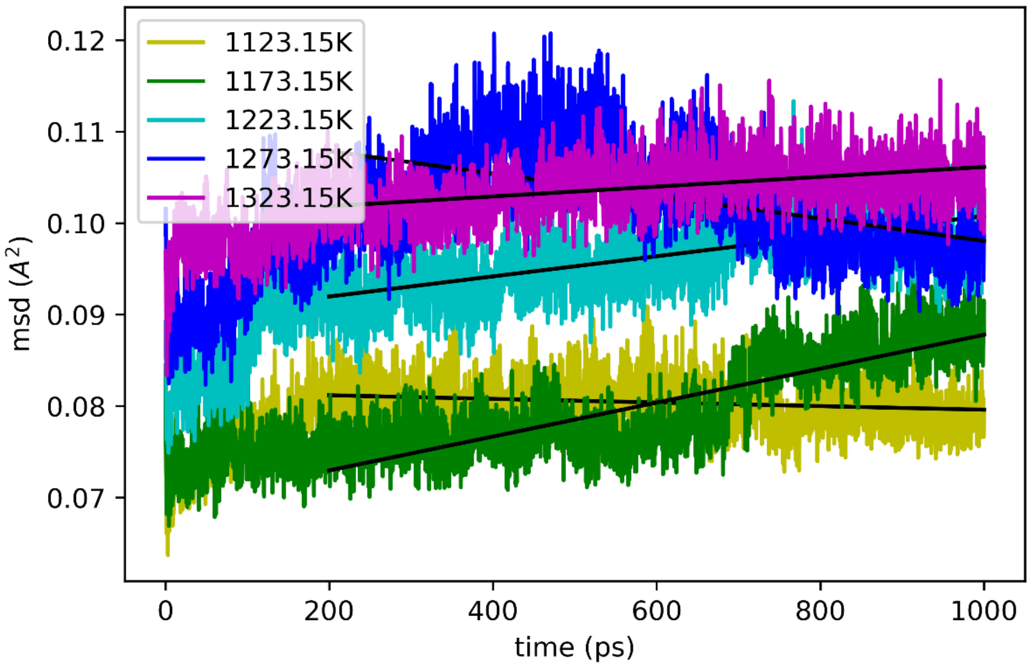

Figure S3 – MSD over time for indium atoms in the ZrO_2(111) over In_2O_3(111) physical mixture with no initial defects

Figure S4 shows the fitting of a linear function to the values of MSD of oxygen atoms from the In_2O_3 lattice over time simulated for the ZrO_2(211) over In_2O_3(111) physical mixture with no initial defects. Curves for temperatures 1123.15 K and 1173.15 K showed a somewhat linear shape since the begging of the measurement and thus were given the standard fitting from 200 ps to 1000 ps. Curves for temperatures 1223.15 K, 1273.15 K and 1323.15 K only began to show a linear shape later in the simulation, and thus were fitted from 500 ps to 1000 ps, 400 ps to 1000 ps and 500 ps to 1000 ps, respectively. It can be seen that the curves for the 3 highest temperatures show almost no slope, which made calculating the activation energy for In_2O_3 oxygens diffusion problematic for this physical mixture.

Figure S5 shows the graph for ln(D) over T^{-1} for this same physical mixture. It is seen that the calculated diffusion activation energy for In_2O_3 oxygen atoms does not carry a physical meaning, and thus was not considered a valid result.

This type of problem happened for all the physical mixtures not shown in the results of the main paper, namely: ZrO_2(211) over In_2O_3(111), ZrO_2(111) monolayer over In_2O_3(111) and ZrO_2(111) monolayer over In_2O_3(111).

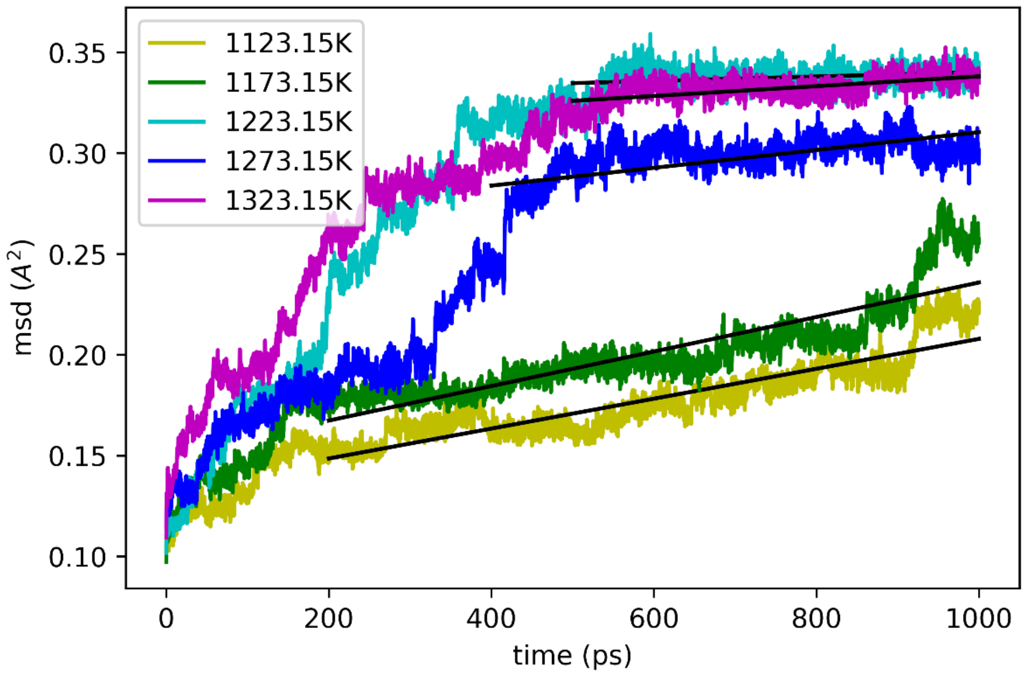

Figure S4 - MSD over time for In_2O_3 oxygen atoms in the ZrO_2(211) over In_2O_3(111) physical mixture with no initial defects

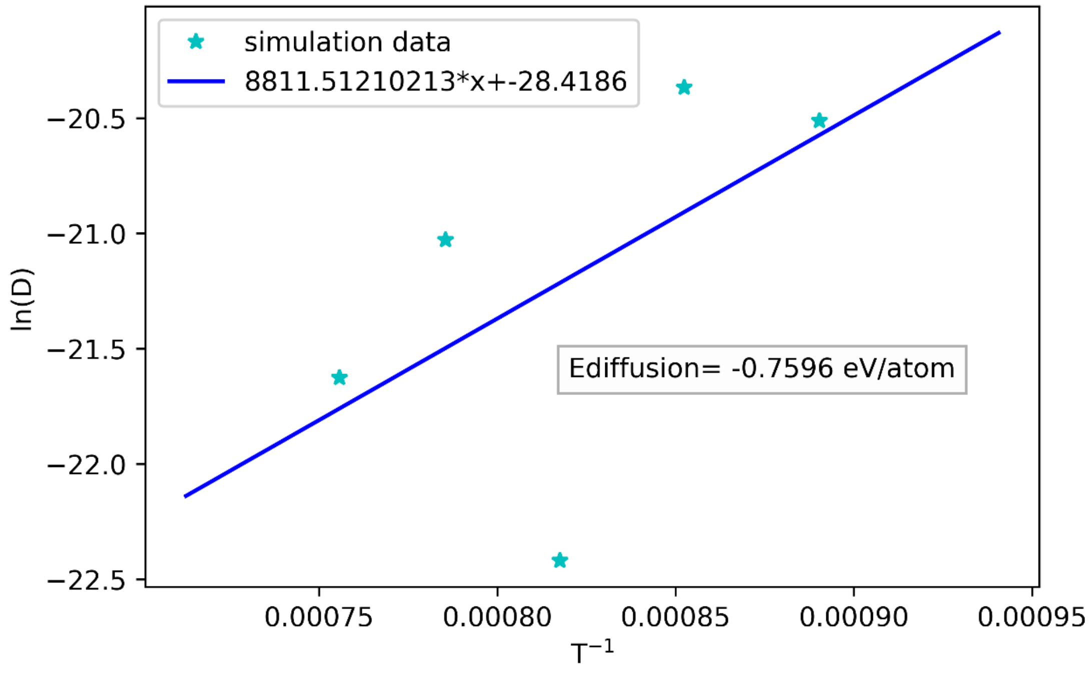

Figure S5 – ln(D) over T^{-1} for In_2O_3 oxygen atoms in the ZrO_2(211) over In_2O_3(111) physical mixture with no initial defects
